# Evaluation of Maternal Dietary n-3 LCPUFA Supplementation as a Primary Strategy to Reduce Offspring Obesity: Lessons From the INFAT Trial and Implications for Future Research

**DOI:** 10.3389/fnut.2020.00156

**Published:** 2020-09-15

**Authors:** Dorothy Marie Meyer, Christina Brei, Bernhard Lorenz Bader, Hans Hauner

**Affiliations:** ^1^Institute of Nutritional Medicine, School of Medicine, Technical University of Munich, Munich, Germany; ^2^ZIEL-Institute for Food and Health, Freising, Germany; ^3^Else Kröner-Fresenius-Center for Nutritional Medicine, Technical University of Munich School of Life Sciences Weihenstephan, Freising, Germany

**Keywords:** childhood obesity, n-3 LCPUFA supplementation, maternal diet intervention, fetal programming, early obesity prevention, breast milk

## Abstract

Preclinical research suggests that early exposure to LCPUFAs is associated with offspring health outcomes, although evidence in humans is rather unclear. In 2006, we established the *Impact of Nutritional Fatty acids during pregnancy and lactation on early human Adipose Tissue development* (INFAT) study, a prospective randomized controlled intervention trial that examined whether decreasing the n-6/n-3 LCPUFA ratio during pregnancy and lactation influences offspring adipose tissue development in children up to 5 years. Our results indicate that maternal supplementation with n-3 LCPUFAs does not reduce offspring obesity risk, which is in line with recent publications. This perspective describes the challenges and lessons learned from our clinical trial. We discuss key findings and critically evaluate differences in study design, methodology, and analyses across similar intervention trials that may partly explain heterogeneous results. Summarizing evidence from human trials, we conclude that n-3 LCPUFA supplementation should not be recommended as a primordial strategy to prevent childhood obesity. Instead, it remains unknown whether n-3 LCPUFA supplementation could benefit high-risk subgroups and some vulnerable maternal/child populations. The perspectives offered herein are derived largely from insights gained from ours and similar n-3 LCPUFA intervention trials and help to provide direction for future research that examines the impact of maternal nutritional exposure on offspring health and disease outcomes.

## Introduction

It is now well-established that perinatal exposure to environmental conditions can influence long-term health. This concept, known as *fetal programming*, has its origins in epidemiological studies performed by Dr. David Barker (1) and was further developed into the framework known as the “Developmental Origins of Health and Disease” (DOHaD) (2). The theory maintains that exposure to adverse environmental stimuli during critical developmental periods can permanently alter regulatory functions and metabolic homeostasis in early life, increasing the risk of chronic diseases later on (3). “Adverse stimuli” also include exposure to a nutrient-excess environment *in utero*. Observational studies explored whether maternal obesity and gestational weight gain influenced offspring health outcomes. Research demonstrated that children born to mothers with obesity were at higher risk for obesity themselves early in life (4, 5), with some showing an elevated risk over their entire life-course (6). The DOHaD hypothesis also gave rise to intervention studies that focused on primordial prevention of obesity by optimizing the maternal diet during pregnancy and lactation (7).

Changes in dietary patterns have led to a shift in the dietary ratio of n-6/n-3 LCPUFA intake in industrialized countries worldwide, favoring foods rich in n-6 LCPUFA sources. Evaluating the effects of changes in fatty acid intake in pregnant women is particularly important, as experimental studies indicate that fetal exposure to dietary LCPUFAs can modulate metabolic pathways involved in gene expression, cell differentiation, and epigenetic modifications (8). Other experimental work provided evidence that arachidonic acid (AA, 20:4n-6) enhances fat cell differentiation at the preadipocyte stage (8, 9), mediated by AA-derived prostacyclin (9, 10). In contrast, eicosapentaenoic acid (EPA, 20:5n-3) and docosahexaenoic acid (DHA, 22:6n-3) confer inhibitory action on fat cell differentiation in a highly coordinated and active process through several pathways (11). Consequently, it was suggested that modifying the composition of dietary fatty acids in pregnancy and lactation could reduce obesity risk in offspring via a programming effect (12). This assumption was mainly based on studies in rodents (13), whereas evidence from human data was largely indirect (14).

Examining the relationship between early exposure to LCPUFAs and childhood obesity gave rise to the “proof of concept” study *Impact of Nutritional Fatty acids during pregnancy and lactation on early human Adipose Tissue development* (INFAT). From 2006 to 2014, we conducted the first human intervention trial that examined whether reducing the maternal n-6/n-3 LCPUFA ratio during pregnancy and lactation influences offspring adipose tissue development and therefore could be used as an early obesity prevention strategy (15–17). We achieved this by supplementing women with 1.2 g of n-3 LCPUFAs (180 mg EPA and 1,020 mg DHA as well as 9 mg vitamin E as an antioxidant) daily and reducing their dietary AA intake to the recommended range of 50–90 mg per day. This report presents a review of key findings. We present INFAT in the context of similar intervention trials and critically evaluate differences in design and methodology that could account for inconsistent findings. Synthesizing insights gained from our study and others, we reassess whether evidence has borne out that early exposure to LCPUFAs influences obesity risk in childhood. Finally, we discuss several promising n-3 LCPUFA intervention strategies that focus on sub-groups and high-risk populations that could improve health outcomes for both mother and child.

## Lessons Learned From the INFAT Study: Implications for Practice

The INFAT study was designed as an open-label, randomized, controlled longitudinal intervention trial, approved by the Technical University of Munich Ethics Committee and is registered in ClinicalTrials.gov: ID NCT00362089.

**Figure 1:**
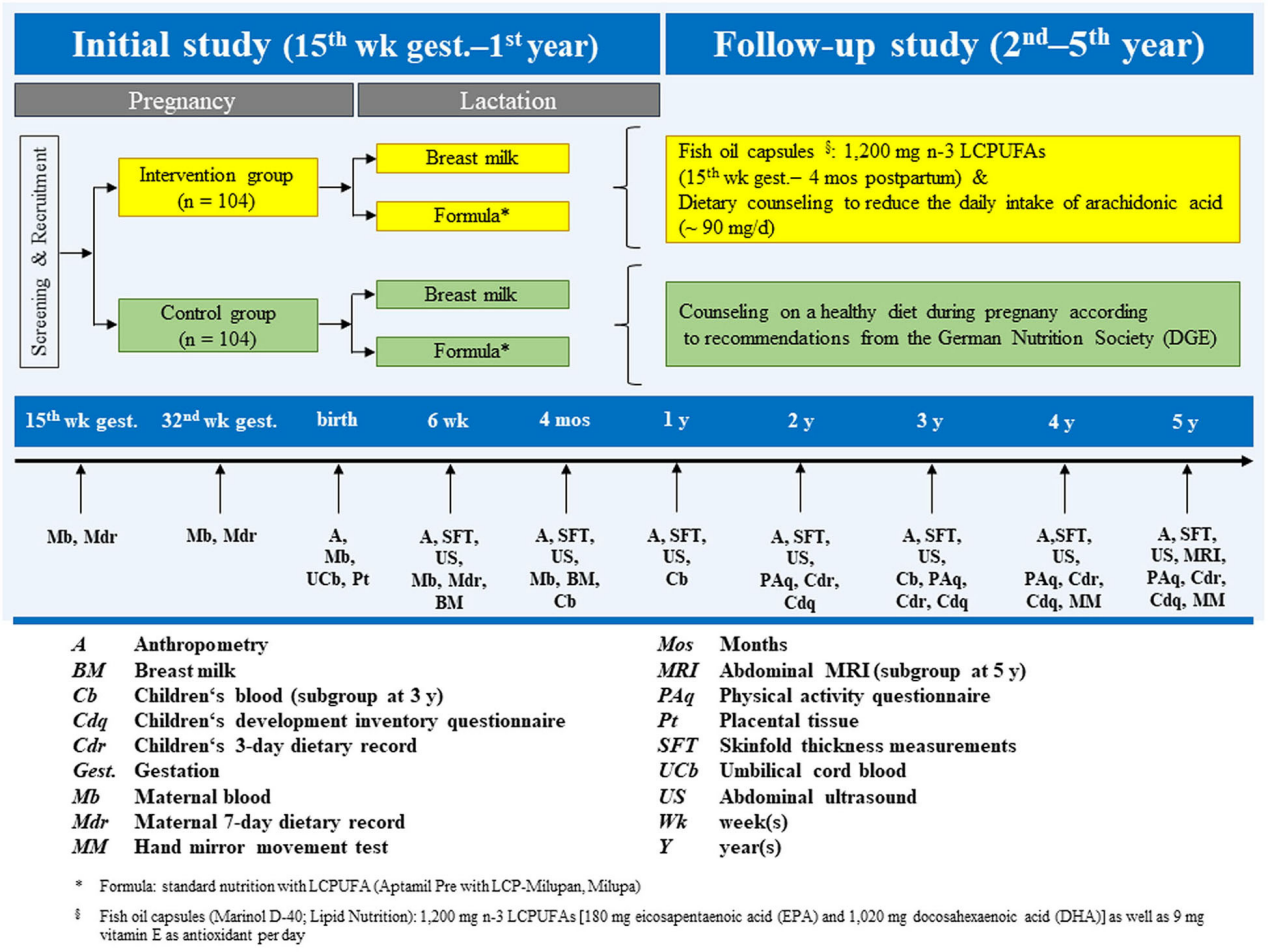
INFAT study design, timeline, and overview of measurements and variables.

A total of 208 women were recruited to participate in the study and randomized to a control (*n* = 104) or intervention (*n* = 104) group. The primary endpoint was the sum of four skinfold thickness (SFT) measurements in infants, assessed four times in the 1st year of life (15, 16). A planned follow-up trial continued annually in offspring from 2 to 5 years old (17). An overview of the study, including the design and data collection at each time-point from recruitment through 5 years, can be seen in Figure 1. A more detailed description of the study design and methods can be found in the respective publications.

Table 1 presents a description of the main findings of the primary and secondary outcomes, listed by topic. In this manuscript, we will focus primarily on the objectives of the initial and follow-up studies, which are the key findings in the first two publications in Table 1 under the heading *Body Composition in Newborns, Infants, and Children*. However, we also made use of our extensive data set by pooling the intervention and control groups to explore relationships between perinatal dietary macronutrient and fatty acid exposure and offspring body composition (18–20, 23). These results are summarized in Table 1.

**Table 1:** Summary of INFAT publications by topic.

**Main outcome measures**	**Study type**	**Age at analysis**	**Main predictors and methods**	**Key findings**	**Reference**
**Body Composition in Newborns, Infants, and Children**					
A, SFT, US	Intervention vs. control group	Birth, 6 wk, 4 mos, and 1 y	Maternal 7-day dietary records^a^^,^^b^ and maternal blood fatty acid profile^a^^,^^b^	Good maternal compliance of fish oil supplementation with the intervention group (assessed by capsule protocol, dietary records, and red blood cell FA analysis). Prolonged gestational duration in the intervention group by ~5 days. No evidence that n-3 LCPUFA supplementation in pregnancy and lactation affects offspring adipose tissue development or distribution.	(16)^§^
A, SFT, US, MRI	Intervention vs. control group	2, 3, 4, and 5 y	Children's physical activity questionnaires and dietary records^f^^,^^g^^,^^h^	No evidence that a dietary reduction of the maternal n-6/n-3 LCPUFA ratio has long-term effects on body composition in preschool children from 2 to 5 years. Influencing factors (physical activity, diet) could be excluded as confounding factors.	(17)^§^
A, SFT, US	Intervention vs. control group and pooled cohort	Birth, 6 wk, 4 mos, and 1 y	Fatty acid profile in maternal blood^a^^,^^b^ and UCB^c^	Maternal n-3 LCPUFAs and n-6 LCPUFAs at 32 weeks' gestation were associated with birth weight and birth length. N-6 LCPUFAs were inversely related to BMI and ponderal index, but not fat mass, in the first year of life.	(18)
A, SFT, US	Intervention vs. control group and pooled cohort	Birth, 6 wk, 4 mos, and 1 y	Breast milk fatty acid profile^d^^,^^e^	The intervention was reflected in the breast milk FA profile; a positive relationship between the FA composition of breast milk and maternal blood was found. Early breast milk was related to the sum of four SFTs at 1 year.	(19)
A, SFT, US, MRI	Pooled cohort	2, 3, 4, and 5 y	Maternal^a^^,^^b^, UCB^c^, and breast milk^d^^,^^e^ fatty acid profile	LCPUFAs in maternal plasma and UCB were not related to child outcomes. Increased breast milk n-3 LCPUFAs predicted several body composition measurements at 2 and 4 years. Breast milk n-6/n-3 fatty acid ratio was inversely related to lean body mass at 4 and 5 years. Overall results do not provide sufficient evidence that LCPUFAs in maternal blood, UCB, and breast milk predict offspring adiposity up to 5 years.	(20)
A, SFT, US	Pooled cohort	6 wk, 4 mos, and 1 y	Adipose tissue measurement methods	Ultrasound was confirmed as a reliable method to estimate fat development and distribution in infants. Subcutaneous and preperitoneal abdominal fat grew at different rates, with an increase in preperitoneal fat in the first year, whereas subcutaneous fat areas increased from 6 weeks to 4 months, and decreased at 1 year postpartum. Sex-specific differences were observed for girls, with greater subcutaneous fat areas from 6 weeks onwards.	(21)
A, SFT, US, MRI	Pooled cohort	2, 3, 4, and 5 y	Adipose tissue measurement methods	Preperitoneal fat steadily increased in boys and girls over childhood, with larger volumes noted in girls from 3 years onwards. Subcutaneous fat decreased in both sexes at 2 years, then slightly increased from 2 to 5 years, with significantly larger volumes measured in girls at any timepoint investigated.	(22)
A, SFT, US, MRI	Pooled cohort	Birth, 1, 3, and 5 y	Maternal 7-day dietary records^a^^,^^b^	Dietary macronutrient intake during early gestation had no impact on offspring body composition. Maternal dietary fat at 32 weeks was inversely related to body composition at birth and 5 years. Higher carbohydrate and sugar intake in late pregnancy were associated with adiposity outcomes at 1 and 3 years.	(23)
**Neurological Development in Children**					
Neurological tests	Intervention vs. control group	4 and 5 y	UCB LCPUFAs^c^, child development inventory^g^^,^^h^, hand mirror movement test^g^^,^^h^	N-3 LCPUFA supplementation in pregnancy and lactation is largely unrelated to neurological development at 4 and 5 years. Some weak associations were observed between UCB fatty acids and improved cognitive outcomes.	(24)
**Placental Gene Expression**					
A, SFT, US	Intervention vs. control group (subpopulation analysis) and pooled subpopulations	Birth, 6 wk, and 1 y	Placental tissue^c^, gene expression analyses by DNA microarray analysis, and RT-qPCR	N-3 LCPUFA intervention in pregnancy has a more pronounced effect on female gene expression, especially in relation to the cell cycle and its associated modulator pathways. Significant changes in mRNA expression for CDK6, PCNA, and TGFN1 were observed in intervention group placentas. CDK6 and PCNA mRNA levels correlated with offspring birth weight and birth weight percentiles. N-3 LCPUFA responsive changes in placental gene expression do not influence offspring body composition.	(25)
**Maternal and Cord Blood Biomarkers**					
A, SFT, US	Pooled cohort	Birth, 6 wk, 4 mos, 1 and 2 y	Maternal insulin, triglycerides, and HOMA-IR^1, 2^; UCB insulin^c^	Maternal metabolic markers were transiently related to child outcomes, but relationships were not consistently significant. Cord blood insulin was inversely related to weight gain up to 2 years, and this relationship was significant and stronger in girls only.	(26)
A, SFT, US, MRI	Pooled cohort	3, 4, and 5 y	Maternal insulin, triglycerides, and HOMA-IR^a^^,^^b^, UCB insulin^c^	Maternal markers of glucose and lipid metabolism were unrelated to child outcomes. Lower cord blood insulin resulted in higher weight gain in girls at 5 years, but with very small effect sizes.	(27)
A, SFT	Intervention vs. control group and pooled cohort	Birth, 6 wk, 4 mos, 1 and 2 y	Leptin, free leptin index, and soluble leptin receptor in maternal blood^a^^,^^b^^,^^d^^,^^e^ and UCB^c^	No effect of the intervention on maternal or cord blood leptin axis. Pooled analysis showed that maternal leptin at 32 weeks' gestation was inversely related to several body composition and growth outcomes at 2 years, while cord blood leptin was positively associated with child outcomes at birth and 2 years. Greater impact was detected for cord blood leptin.	(28)
A, SFT, US, MRI	Pooled cohort	3, 4, and 5 y	Leptin in maternal blood^b^ and UCB^c^	Maternal leptin was inversely associated with growth in early childhood. Cord blood leptin was inversely and transiently related to certain growth and adipose tissue development indices in young children. Effect sizes were very small.	(29)
A, SFT	Pooled cohort	6 wk, 4 mos, 1 and 2 y	Leptin and adiponectin in maternal blood^d^^,^^e^ and breast milk^d^^,^^e^	Breast milk adiponectin was associated with increased fat mass and weight gain up to 2 years.	(30)
A, SFT	Pooled cohort	3, 4, and 5 y	Leptin and adiponectin in breast milk^d^^,^^e^	Breast milk leptin and adiponectin were largely unrelated to body composition in children up to 5 years.	(31)
A, SFT, US, MRI	Pooled cohort	Birth, 1, 2, 3, 4, and 5 y	HMW and total adiponectin in UCB^c^ and child plasma^f^	Neither HMW nor total adiponectin in cord blood were found to predict body composition in early childhood up to 5 years. No associations between HMW or total adiponectin, measured in child plasma at 3 years, and child growth or fat development at 3, 4, or 5 years were observed.	(32)

§ Publications with primary outcome analyses

a 15 weeks' gestation,

b 32 weeks' gestation,

c delivery,

d 6 weeks postpartum,

e 4 months postpartum,

f 3 years,

g 4 years,

h *5 years*.

*Pooled cohort analyses: no evidence of a difference in analyzed parameters between intervention and control group was found. Hence, the two groups were pooled for further analysis*.

*A, Anthropometrics (e.g., body weight, body length or body height, BMI (body mass index) percentiles, head circumference, arm circumference, waist circumference); UCB, umbilical cord blood; HMW, high molecular weight; HOMA-IR, homeostatic model assessment of insulin resistance; FA, fatty acids; LCPUFA, long chain polyunsaturated fatty acids; mos, months; MRI, subcutaneous and visceral (cm^3^) adipose tissue development by magnetic resonance imaging (subgroup) at 5 years; pp, postpartum; RT-qPCR, quantitative reverse transcription polymerase chain reaction; SFT, adipose tissue development by sum of four skinfold thicknesses; US, subcutaneous and preperitoneal (mm^2^) adipose tissue development by ultrasound from 6 wk pp onwards; wk, weeks; y, years*.

A secondary interest was to identify biomarkers that could predict adiposity outcomes. To this end, we pooled our cohorts to investigate several metabolic and hormonal factors in maternal blood, cord blood, and breast milk, including adiponectin, leptin, and indices of insulin resistance (26–32). A description of findings from all secondary analyses is found in Table 1 (15–17).

The long-term follow-up afforded us the opportunity to observe that several relationships between perinatal determinants and offspring body composition change over time. For example, while significant associations between n-3 and n-6 LCPUFAs, leptin, and adiponectin in maternal blood, cord blood, and breast milk were present early in life (19, 28, 30), they did not persist up to 5 years (20, 29, 31, 32), as summarized in Table 1. This is an important observation to consider in planning future longitudinal studies, as around 5 years is known as the age of adiposity rebound and is thought to be a critical age that can predict later obesity (33). Recent findings from a German population study confirmed this link by reporting that accelerated weight gain in the preschool years is predictive of overweight and obesity in adolescence (34).

Most importantly, our results did not indicate that reducing the ratio of n-6/n-3 LCPUFAs during pregnancy and lactation influences adipose tissue development in early childhood, arguing against its use as an effective strategy to reduce obesity risk (15–17). We also found no evidence that supports maternal n-3 LCPUFA supplementation to improve offspring cognitive outcomes (24) (Table 1).

Many randomized controlled trials have assessed early childhood growth indirectly, using height and weight (35), BMI *z*-scores (36), and waist circumference (36) to estimate body composition. INFAT comprises one of the largest data sets of body composition and fat distribution measurements in children from birth to 5 years. An important contribution to the research community from INFAT was our assessment of body fat patterning and accretion via abdominal ultrasound throughout early childhood. We were the first to demonstrate that abdominal ultrasound was a reliable and reproducible method in preschoolers for measuring subcutaneous and preperitoneal fat (21, 22), the latter considered an approximation of visceral fat in children (37). Major advantages of ultrasound over other imaging techniques, such as MRI and dual-energy X-ray absorptiometry, are its portability and lower cost-per-examination, which make ultrasound a practical tool for measuring body fat in the clinical setting.

Estimating fat depots by abdominal ultrasound revealed that subcutaneous and preperitoneal fat develop at different rates and volumes in boys and girls (21, 22). INFAT girls had significantly larger measurements of subcutaneous fat than boys from birth up to 5 years, while differences in preperitoneal fat were first detectable from 3 years onward, with girls showing larger areas up to age 5. Concerning sex-specific effects and the importance of the placenta for fetal programming and growth, we revealed sexually dimorphic gene expression and n-3 LCPUFA responsiveness, with more pronounced effects in female placentas, especially in relation to the cell cycle (25). Analyzed placental gene expression correlated with birth weight but not with offspring adipose tissue growth in the first year (see description in Table 1). Our results underscore the importance of performing analyses to detect sex-specific differences and gain new insights into underlying mechanisms and sex-specific offspring growth.

## Critically Appraising the INFAT Research Design and Methodology: Challenges and Recommendations for Future Studies

We assessed adherence to the fish oil capsule regimen and reduction of meat products with capsule protocol diaries and 7-day dietary records, respectively. Compliance was further confirmed by measuring the maternal fatty acid profile in blood and breast milk at four-time points during the study. Significant differences in LCPUFA levels between the intervention and control groups were observed in all maternal and infant tissues measured (18, 19), including plasma phospholipids, erythrocyte membranes, placenta, umbilical cord blood, and breast milk. There was also a reduced intake of AA from meat products and other foods in the intervention group at 32 weeks' gestation compared to the control group (129.8 ± 76.6 vs. 160.6 ± 80.6 mg AA/d, *p* < 0.001) (16). Overall, the n-6/n-3 LCPUFA ratio in the maternal diet was around 3.5:1 in the intervention and 7:1 in the control group (16), which was the ratio we hoped to achieve in the intervention group. Nevertheless, we were unable to confirm that achieving this ratio in pregnancy and lactation affects the accretion or distribution of adipose tissue in young children (16, 17). Our results are in line with two systematic reviews and a recent meta-analysis (38–40). Importantly, studies included in these analyses were very heterogeneous, both in investigative methods and primary outcomes, which raises questions as to whether differences in study design and analytical strategies may partly explain the lack of effects.

To our knowledge, we are the only research group whose primary aim was to balance the n-6/n-3 LCPUFA ratio by supplementing n-3 LCPUFAs while concomitantly decreasing the maternal dietary intake of n-6 LCPUFAs. Most interventions have focused solely on increasing exposure to perinatal n-3 LCPUFAs (40), which is worth discussing since a high background n-6 LCPUFA status limits the incorporation of n-3 LCPUFAs in phospholipids (41). Moreover, it is known that n-6 fatty acids can have a pro-adipogenic effect (9) and research indicates that maternal n-6 LCPUFA levels are strongly associated with offspring adiposity outcomes (42). This evidence suggests that studies with larger cohorts are needed to further explore whether clinically meaningful effects can be detected in offspring after reduced exposure to n-6 LCPUFAs *in utero*.

An important shortcoming of INFAT is that it was not adequately powered at the 5-year follow-up, which weakened our overall findings. The power calculation of 80% was based on a sample size that accounted for a drop-out rate of 30% at 12 months postpartum. We received additional funding during the first year to continue the follow-up period to 5 years postpartum. Notably, the total number of children for whom we had SFT measurements at 5 years was 114, or 55% of the original sample. The sizeable attrition rate between 1 and 5 years can be partially explained because several families chose to drop-out after the initial study, despite the considerable effort made by INFAT researchers to encourage continued participation. Loss to follow-up is common in longitudinal studies, and ours was no exception. A pediatric obesity prevention study investigated reasons for attrition and found that overweight/obesity and related factors were the most salient determinants (43), a relevant finding for INFAT given that our primary outcome was adipose tissue mass measurements. Our experience with high drop-out rates emphasizes the need to clearly communicate intervention aims and follow-up duration with study participants at the onset of longitudinal studies.

Notably, when comparing perinatal intervention studies with similar aims, there are considerable differences in the dosage and composition of LCPUFAs, with little consensus on the optimal intake amount. The European Food and Safety Authority (EFSA) recommends a daily intake of 250 mg DHA + EPA for adults, with an additional 100–200 mg of DHA during pregnancy and lactation (44). Dosage in intervention studies has ranged from 800 mg to 4 g of n-3 LCPUFAs per day and is mainly composed of DHA and EPA, usually with higher proportions of DHA (40, 45). We were concerned about the potential adverse effects of higher n-3 LCPUFA doses and selected 1.2 g of n-3 LCPUFAs (180 mg EPA and 1,020 mg DHA) as a compromise between efficacy and safety. Given the high variability in dosage and general lack of effects, it is possible that the dose of total LCPUFAs or the specific n-3 LCPUFA composition was not high enough to observe the intended effect. Since designing our study, preclinical research has explored how different proportions of EPA and DHA in n-3 supplements have varying effects on inflammatory pathways. Evidence shows that inflammatory markers are decreased at EPA/DHA ratios of 1:1, 1:2, and 2:1 (46, 47), with the most pronounced effects observed at the ratio of 2:1 (47). Dasilva et al. (46) observed that EPA had increased antioxidant and anti-inflammatory capacities compared to DHA, and that n-3 LCPUFAs from fish oil versus linseed or soybean oil was significantly more effective. Taken together, this evidence suggests that in addition to the dose of n-3 LCPUFAs, the proportion of EPA/DHA, ideally from marine sources, is also important.

Another question that remains is whether the baseline maternal LCPUFA levels were already too high pre-intervention. There was considerable variation in the pre-intervention levels of EPA, DHA, AA, and n-6/n-3 LCPUFAs across participants (16). Nonetheless, we did not control for pre-levels in analysis which could have obscured an intervention effect. A recent study by Vinding et al. (45) found that prenatal n-3 LCPUFA supplementation had an overall effect on offspring somatic growth at 6 years after controlling for baseline maternal n-3 LCPUFA levels. However, it is interesting that they did not observe an interaction effect between maternal pre- and post intervention n-3 LCPUFA levels on child body composition (45).

There is also some debate as to whether women with high baseline n-3 LCPUFA levels should be excluded from intervention trials. A review that examined the efficacy of n-3 LCPUFA supplementation on reducing cardiac mortality concluded that there may be a threshold pre-intervention level in which n-3 LCPUFAs no longer have an effect (48). Given these findings, future LCPUFA intervention studies should consider routinely measuring baseline concentrations and statistically controlling for pre-intervention levels. Moreover, more research is needed to understand whether baseline n-3 LCPUFA concentrations modify relationships between n-3 LCPUFA supplementation and child outcomes (10, 49–51).

It is worthwhile to note that most studies began their intervention in the second trimester of pregnancy (38–40). Evidence of human adipocyte development has been observed between the 14th and 16th week of gestation (52), which supports the INFAT intervention start-time of ~15 weeks' gestation. However, maternal nutrition in the first trimester is critical for the growth and development of certain vital fetal organs (53). Early changes in maternal LCPUFA levels could have a programming effect on offspring, and some researchers have questioned whether an intervention should start earlier in pregnancy (54). First trimester interventions would entail recruiting women in the preconception period, which could be achieved by engaging primary care providers to actively participate in developing and implementing perinatal intervention trials.

## Direction for Future Research: Targeting LCPUFA Interventions for High-Risk Populations

Despite preclinical evidence demonstrating links between perinatal nutrition and an increased risk of offspring obesity and related diseases (13), research in humans has been less convincing. Most of the supporting evidence has been derived from epidemiological studies. Importantly, this relationship is often observed in circumstances of extreme under- or over-nutrition, such as offspring of mothers who survived the Dutch Famine (1944–1945) (55, 56), which calls into question whether non-extreme alterations in perinatal nutrient availability are causally implicated in the development of chronic obesity-related diseases, or if other exposures *in utero* and early life are at play. Thus far, evidence that perinatal LCPUFA supplementation ameliorates offspring obesity outcomes in humans is largely unsubstantiated (38–40). However, it is unclear whether certain high-risk subgroups could benefit from n-3 supplementation and insights gained from the INFAT study could help shape new directions for research.

There is strong evidence that supports prenatal n-3 LCPUFA supplementation to reduce the incidence of preterm birth (16, 57–60), especially in high-risk pregnancies (60, 61). A recent study of 184 countries reported that countries with low n-3 LCPUFA dietary intake have a higher overall incidence of preterm births (62). Importantly, the researchers observed a threshold benefit to n-3 LCPUFAs, where no associations were observed between preterm birth incidence and countries whose dietary intake was above 600 mg/day. Over 95% of American childbearing-aged women do not meet the current recommendation of 250 mg n-3 LCPUFAs per day (63), with similar findings reported in Europe (64). Research has shown that a total percentage of ≤4.1% of n-3 fatty acids in maternal blood in early pregnancy strongly predicts preterm birth (65). These findings provide evidence that support n-3 LCPUFA interventions in populations whose daily consumption of n-3 LCPUFAs falls short of the recommended intake, or for women with high-risk pregnancies (61, 65–67). Based on the aforementioned multi-country study by Ciesielski et al. (62), it is also worth considering revising the daily recommendation of n-3 LCPUFAs to 600 mg during pregnancy to decrease the risk of preterm births, estimated to be 6.2% and 10.6% of all live births in Europe and North America, respectively (62, 68).

Another opportunity for targeted LCPUFA interventions is investigating outcomes in women with obesity. INFAT was designed as a concept validation study, therefore women with underweight or obesity were excluded. INFAT children were also leaner than their peers at 5 years, with only one child (~1%) having obesity, a marked contrast to the German pediatric population with obesity rates around 3.3–5.4% at this age (69). N-3 LCPUFAs are known to exhibit anti-inflammatory and anti-obesogenic properties in animals (70), although data in humans do not consistently confirm these associations. One study that provided DHA supplements to pregnant women with obesity or gestational diabetes found no significant effects on offspring adiposity outcomes (71). More data are needed to determine whether supplementation in pregnant women in higher BMI categories may be a promising intervention to reduce offspring obesity risk.

## Conclusion

Findings from the INFAT study demonstrate that perinatal administration of n-3 LCPUFAs has only transient effects in early childhood, arguing against this dietary intervention as a strategy for the primary prevention of childhood obesity. Instead, research aims should shift toward investigating whether certain vulnerable and high-risk subgroups could benefit from n-3 LCPUFA interventions. Insights gained from INFAT and other studies discussed herein point to promising results and implications for planning future intervention studies. Irrespective of our study results, long-term follow-up studies are necessary to understand how and to what extent perinatal nutrition and consecutive changes of regulatory hormonal systems influence health and disease risk in childhood and, ultimately, over the life course.

## Data Availability Statement

The original contributions presented in the study are included in the article/supplementary material, further inquiries can be directed to the corresponding author/s.

## Ethics Statement

The studies involving human participants were reviewed and approved by Technical University of Munich Ethics Committee. The patients/participants provided their written informed consent to participate in this study.

## Author Contributions

HH designed the study and supervised the project. DM wrote the paper with input from all authors. BB, CB, and DM designed the table and figure. All authors discussed the findings and contributed to the manuscript revision. All authors contributed to the article and approved the submitted version.

## Conflict of Interest

The authors declare that the research was conducted in the absence of any commercial or financial relationships that could be construed as a potential conflict of interest.
